# Analysis of SARS-CoV-2 in Nasopharyngeal Samples from Patients with COVID-19 Illustrates Population Variation and Diverse Phenotypes, Placing the Growth Properties of Variants of Concern in Context with Other Lineages

**DOI:** 10.1128/msphere.00913-21

**Published:** 2022-05-02

**Authors:** Tessa Prince, Xiaofeng Dong, Rebekah Penrice-Randal, Nadine Randle, Catherine Hartley, Hannah Goldswain, Benjamin Jones, Malcolm G. Semple, J. Kenneth Baillie, Peter J. M. Openshaw, Lance Turtle, Grant L. Hughes, Enyia R. Anderson, Edward I. Patterson, Julian Druce, Gavin Screaton, Miles W. Carroll, James P. Stewart, Julian A. Hiscox

**Affiliations:** a Institute of Infection, Veterinary and Ecological Sciences, University of Liverpool, Liverpool, United Kingdom; b NIHR Health Protection Research Unit in Emerging and Zoonotic Infections, Liverpool, United Kingdom; c Department of Respiratory Medicine, Alder Hey Children’s Hospital, Liverpool, United Kingdom; d The Roslin Institute, University of Edinburgh, Edinburgh, United Kingdom; e National Heart and Lung Institute, Imperial College London, London, United Kingdom; f Departments of Vector Biology and Tropical Disease Biology, Center for Neglected Tropical Diseases, Liverpool School of Tropical Medicine, Liverpool, United Kingdom; g Virus Identification Laboratory, Doherty Institute, University of Melbourne, Melbourne, Australia; h Nuffield Department of Medicine, University of Oxford, Oxford, United Kingdom; i Public Health England, Salisbury, United Kingdom; j Department of Infectious Disease, University of Georgia, Georgia, USA; k A*STAR Infectious Diseases Laboratories (A*STAR ID Labs), Agency for Science, Technology and Research (A*STAR), Singapore; Icahn School of Medicine at Mount Sinai

**Keywords:** SARS-CoV-2, COVID-19, clinical samples, minor genomic variants, growth kinetics, cell culture, coronavirus, RNA sequencing, variants

## Abstract

New variants of SARS-CoV-2 are continuing to emerge and dominate the global sequence landscapes. Several variants have been labeled variants of concern (VOCs) because they may have a transmission advantage, increased risk of morbidity and/or mortality, or immune evasion upon a background of prior infection or vaccination. Placing the VOCs in context with the underlying variability of SARS-CoV-2 is essential in understanding virus evolution and selection pressures. Dominant genome sequences and the population genetics of SARS-CoV-2 in nasopharyngeal swabs from hospitalized patients were characterized. Nonsynonymous changes at a minor variant level were identified. These populations were generally preserved when isolates were amplified in cell culture. To place the Alpha, Beta, Delta, and Omicron VOCs in context, their growth was compared to clinical isolates of different lineages from earlier in the pandemic. The data indicated that the growth in cell culture of the Beta variant was more than that of the other variants in Vero E6 cells but not in hACE2-A549 cells. Looking at each time point, Beta grew more than the other VOCs in hACE2-A549 cells at 24 to 48 h postinfection. At 72 h postinfection there was no difference in the growth of any of the variants in either cell line. Overall, this work suggested that exploring the biology of SARS-CoV-2 is complicated by population dynamics and that these need to be considered with new variants. In the context of variation seen in other coronaviruses, the variants currently observed for SARS-CoV-2 are very similar in terms of their clinical spectrum of disease.

**IMPORTANCE** SARS-CoV-2 is the causative agent of COVID-19. The virus has spread across the planet, causing a global pandemic. In common with other coronaviruses, SARS-CoV-2 genomes can become quite diverse as a consequence of replicating inside cells. This has given rise to multiple variants from the original virus that infected humans. These variants may have different properties and in the context of a widespread vaccination program may render vaccines less effective. Our research confirms the degree of genetic diversity of SARS-CoV-2 in patients. By comparing the growth of previous variants to the pattern seen with four variants of concern (VOCs) (Alpha, Beta, Delta, and Omicron), we show that, at least in cells, Beta variant growth exceeds that of Alpha, Delta, and Omicron VOCs at 24 to 48 h in both Vero E6 and hACE2-A549 cells, but by 72 h postinfection, the amount of virus is not different from that of the other VOCs.

## INTRODUCTION

SARS-CoV-2 emerged in late 2019 in Wuhan, China, and causes COVID-19 (1). This can be a fatal infection with severe immunopathology in the respiratory system (2, 3). The virus has since spread worldwide and resulted in more than five million deaths (4), placing large burdens on health care infrastructures and global economies. Several vaccines are now in use and have resulted in reduced hospitalization rates in countries with large-scale vaccine rollouts. However, multiple variants have been identified worldwide, and these have the potential for an increase in transmissibility and virulence and a decrease in the effectiveness of public health measures, such as testing or vaccine efficacy, leading to the label of variants of concern (VOCs).

SARS-CoV-2 has a single-stranded, positive-sense RNA genome about 30 kb in length. The first two-thirds of the genome encode the viral nonstructural proteins (NSP1 to -16), which includes the viral RNA-dependent RNA polymerase (NSP12). Several viral RNA synthesis processes occur during infection, including replication of the genome and transcription of a nested set of subgenomic mRNAs (sgmRNAs). The latter process requires discontinuous transcription during negative-strand synthesis (5). As a natural consequence, coronaviruses have high levels of recombination. This can result in both deletions and insertions and template switching as well as the formation of defective RNAs. An example of this is the probable insertion of the furin cleavage site in the spike glycoprotein (6). Although SARS-CoV-2 and other coronaviruses have some type of proofreading capability (7), this is generally thought to help maintain their large genomes without entering error catastrophe. Otherwise the accumulation of deleterious mutations would result in a rapid loss of fitness and extinction of a viral population (8). Additionally, potential genome modifications can result from nucleotide changes through the action of cellular proteins involved in RNA processing (9). These drivers of genetic diversity and the numbers of people infected have led to multiple lineages and variants of SARS-CoV-2 being identified worldwide. The nucleotide diversity between these variants is still very narrow compared to the diversity observed in other coronaviruses and their phenotypic consequences (10, 11).

The sgmRNAs encode the main structural proteins, including the envelope protein (E) protein, the membrane (M) protein, the nucleocapsid (N) protein, and the spike (S) glycoprotein. The S protein is a component of the enveloped virion and interacts with the angiotensin converting enzyme-2 receptor (ACE2) found on human cells. The S protein is also the major source of neutralizing epitopes and therefore under selection pressure in coronaviruses (and SARS-CoV-2). Other viral proteins are involved in modulating the innate immune response.

Many variations in the coronavirus genome can be identified in the S gene, as this is a major site for selection pressure (12–14). For SARS-CoV-2, the D614G substitution in the S protein, which emerged by March 2020, demonstrated improved transmissibility compared to Wuhan variants and proceeded to dominate worldwide. This mutation is most often accompanied by another amino acid substitution in NSP12, P323L (15). In September 2020, a variant of concern, B.1.1.7 (Alpha), was detected in the United Kingdom that possessed 23 mutations distinct from the Wuhan reference sequence, including the N501Y substitution in the receptor binding domain of the S protein. This is thought to increase the affinity of spike protein to ACE2 receptor (16). Initial data suggested this variant was associated with an increased risk of hospitalization and death (17). The variant spread to several countries, and modeling studies suggested increased transmissibility (18). Experiments in a hamster model of infection identified increased viral shedding compared to the D614G variant (19). Since November 2021, another highly transmissible variant, known as B.1.1.529 or Omicron variant, has superseded the B.1.617.2 (Delta) variant in many places worldwide.

As variants are likely to continue to emerge on a background of incomplete vaccination globally, understanding the significance of such variants both *in vitro* and *in vivo* is important to provide biological mechanistic data rather than rely on modeling to determine their potential threat to vaccines or whether they have a transmission advantage. To investigate the genetic and phenotypic diversity of SARS-CoV-2 in patients and in the context of the emergence of the Alpha, Beta, Delta, and Omicron VOCs and concerns around potentially higher viral loads, the growth of these variants was benchmarked against a range of clinical isolates from other samples taken during the outbreak representing several different lineages.

## RESULTS

**FIG 1:**
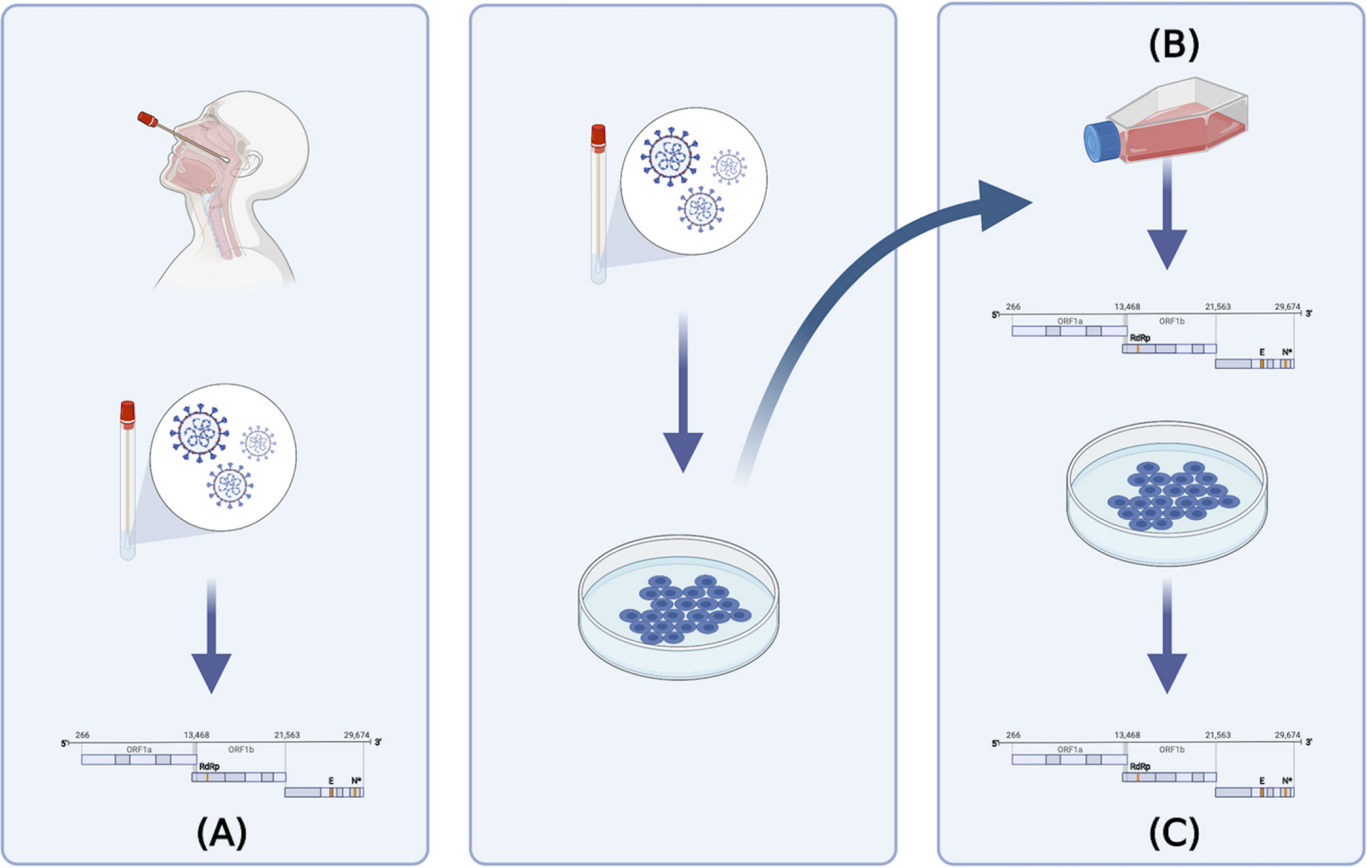
Testing strategy. (A) Nasopharyngeal swabs from patients with COVID-19 recruited to the ISARIC-4C study were sequenced using an amplicon-based approach on the Oxford Nanopore MinION platform (P0). Virus was isolated from the same nasopharyngeal swabs (P1). (B) Viral isolates from the ISARIC-4C study, B.1.1.7, B.1.351, B.1.617.2, B.1.1.529, and Victoria isolates were grown up into stocks that were then sequenced. (C) Viral stocks were titrated and used to infect hACE2-A549 cells, and 72 h postinfection supernatants were sequenced.

**FIG 2:**
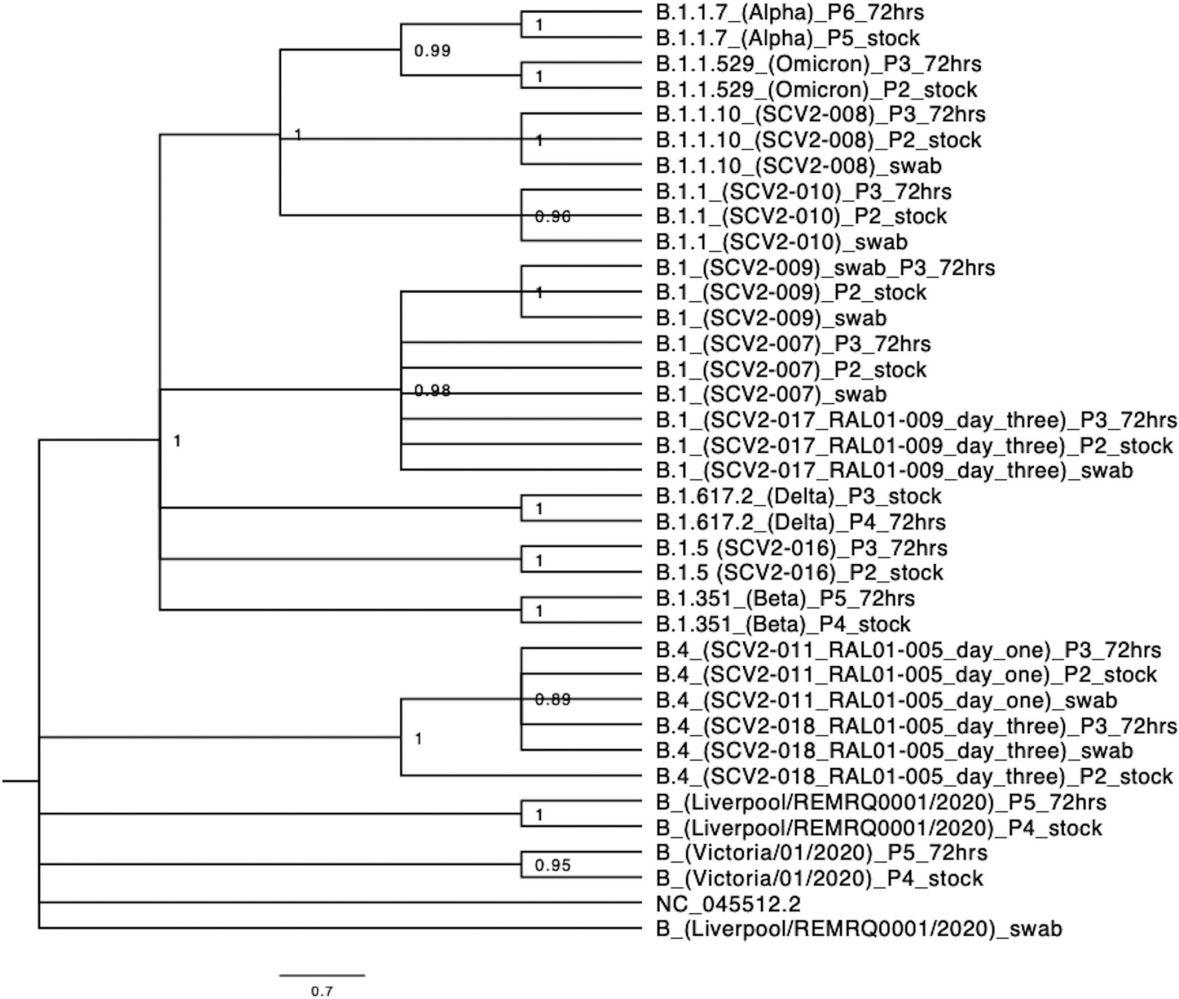
Phylogenetic tree of all variants sequenced. All patient swabs (labeled _swab), stock viruses (labeled _Passage#_stock), and variants after 72 h of growth in hACE2-A549 cells (labeled _Passage#_72hrs) were sequenced and a phylogenetic tree constructed to demonstrate the limited diversity between dominant viral genomes of different variants used in this study.

Although dominant viral genomes for SARS-CoV-2 are reported on global databases from the sequencing of clinical specimens, in reality the virus exists as a population within an individual and may also include defective interfering RNAs. Likewise, in some pipelines, viral genomes or variants containing out-of-place stop codons within ORFs will not be returned as dominant viral sequences even though they may be dominant. Theoretically, minor genomic variants, which could represent 49% of other genomes within the same individual, may impact the viral phenotype. To investigate the sequence diversity of SARS-CoV-2 in a patient and to compare the growth of these viral populations to identified VOCs, nasopharyngeal swabs were taken from patients with COVID-19. These were sequenced and the genotypes and growth of isolated viruses compared in two cell culture models (Fig. 1). A phylogenetic tree of all viruses sequenced in this study is shown in Fig. 2.

### Sequence variation of SARS-CoV-2 in clinical swabs compared to Wuhan reference strain.

**FIG 3:**
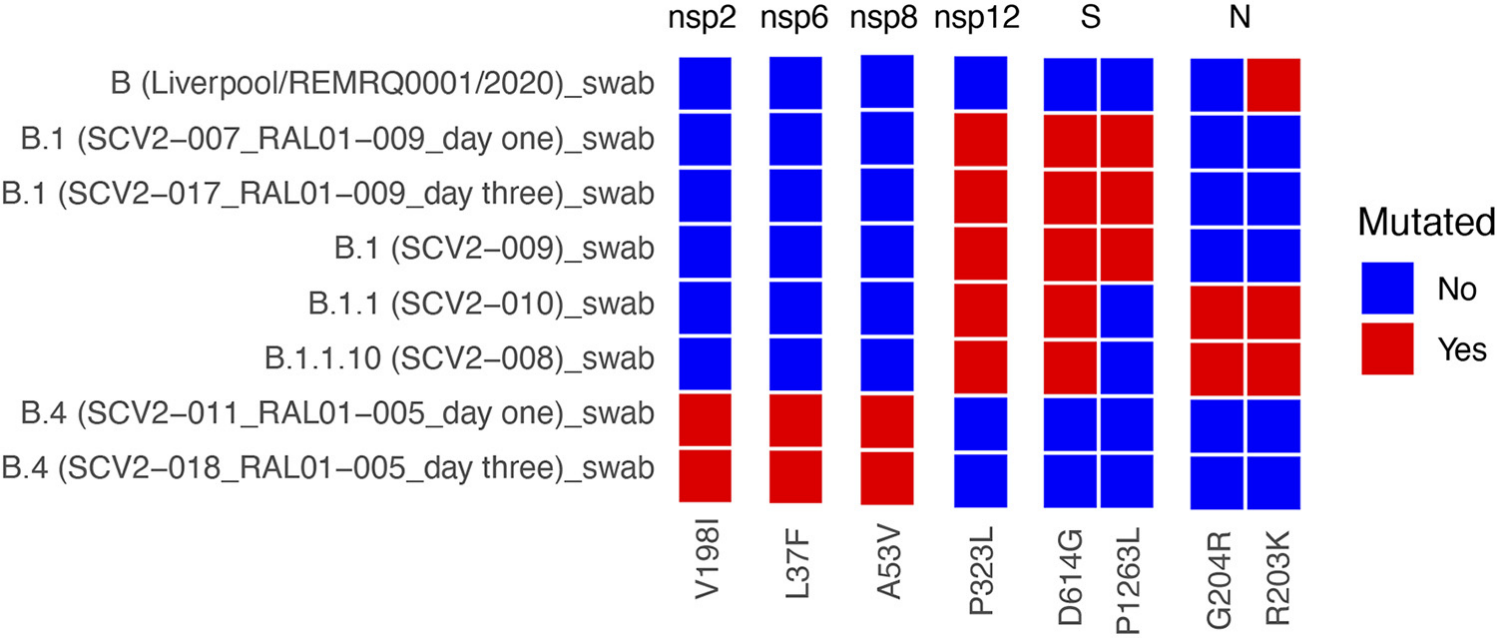
Comparison of the dominant viral genome sequence of SARS-CoV-2 in ISARIC4C swabs collected from patients in hospital with COVID-19 to the Wuhan reference sequence.

To investigate sequence variation, nine swabs from patients representing different time points in the outbreak in the United Kingdom were found to contain recoverable virus (Table 1). The virus population in these swabs was sequenced, and both dominant viral genomes and minor genomic variants were determined. Dominant viral genome sequence variation for each swab was compared to that of the reference genome (GenBank accession no. NC_045512; Wuhan-Hu-1) to determine divergence of the genome, with minor genomic variants listed for the secondary and tertiary positions (see Table S1 in the supplemental material). Most genomes demonstrated a few amino acid variations compared to the reference sequence. For example, Liverpool/REMRQ0001/2020, a lineage B variant, was sequenced from the swab of a patient from the *Diamond Princess* cruise ship (February 2020) and had only one substitution present, a conserved R203K in the N protein, and lacked the D614G mutation in S protein (Fig. 3). In comparison, SCV2-009 (lineage B.1), a virus isolated from a swab sampled in March 2020 (Fig. 3), now possessed the D614G and P323L substitutions in the S protein and NSP12, respectively. These are in contrast to the Alpha variant, which emerged later in 2020 and is characterized by the presence of 23 amino acid differences from the reference genome (20).

**TABLE 1:** List of variants used

Lineage	Virus	ID	Date of onset (mo/day/yr)	Day of sampling	Passage no. of stock
B	Liverpool/REMRQ0001/2020	REMRQ-001	2/23/2020	1	P4
B	Victoria/01/2020	NA	1/2020	NA	P4
B.1	SCV2-009	REMRQ-028	3/23/2020	1	P2
B.1	SCV2-007_RAL01-009_day one	RAL01-009	2/25/2020	1	P2
B.1	SCV2-017_RAL01-009_day three	RAL01-009	2/25/2020	3	P2
B.4	SCV2-011_RAL01-005_day one	RAL01-005	2/25/2020	1	P2
B.4	SCV2-018_RAL01-005_day three	RAL01-005	2/25/2020	3	P2
B.1.1	SCV2-010	RAL01-004	2/24/2020	1	P2
B.1.5	SCV2-016	A1344	6/5/2020		P2
B.1.1.10	SCV2-008	RAL01-018	3/2/2020	1	P2
B.1.1.7	Alpha	NA^*a*^	1/2021		P5
B.1.351	Beta	NA	11/2021		P4
B.1.617.2	Delta	NA			P3
B.1.1.529	Omicron	NA			P2

a NA, not applicable.

10.1128/msphere.00913-21.1 TABLE S1 Dominant viral amino acid changes and minor variant changes found in swabs, stocks, and supernatants from hACE2-A549 cells incubated with virus for 72 h. Download Table S1, XLSX file, 0.01 MB. Copyright © 2022 Prince et al. 2022 Prince et al. https://creativecommons.org/licenses/by/4.0/ This content is distributed under the terms of the Creative Commons Attribution 4.0 International license.

Clinical samples may contain a diverse population of viral genotypes with differing mutations that can lead to founder effect. For example, analysis of the virus population in the nasopharyngeal swab, from SCV2-009, illustrated the variety associated with the virus. Taking the P323L substitution in NSP12 as an example in this sample, out of an amino acid coverage of 202, 170 amino acids mapped to L, 12 to P, and 9 to F. For the D614G substitution in the S protein, out of an amino acid coverage of 3,452, 3,360 amino acids mapped to G, 24 to S, and 21 to V. This general pattern was reflected in SARS-CoV-2 from other nasopharyngeal swabs. For example, in SCV2-010 (lineage B.1.1), in NSP12, out of an amino acid coverage of 285, 273 mapped to L, 50 to I, and 3 to P. In isolate SCV2-008 (lineage B.1.1.10), in NSP12, out of an amino acid coverage of 153, 130 mapped to L, 9 to P, and 7 to F. This suggested, for NSP12, that at the minor genomic variant level the reference sequence amino acid was still present, but other amino acids such as F were common (Table S1). In the clinical swabs for SCV2-008 (B.1.1.10) and SCV2-010 (B.1.1.), for example, in N at position 204, the second most common feature was a premature stop codon. Longitudinal samples taken from two patients were sequenced and grown. Day 1 and day 3 postrecruitment samples from the patients yielded SCV2-011 (B.4) and SCV2-018 (B.4) (patient RAL01-005) and SCV2-007 (B.1) and SCV2-017 (B.1) (patient RAL01-009), respectively. These did not vary at the dominant viral genome sequence level between each other but did at the minor genomic variant level (Table S1).

### Comparison of sequence variation in stocks and after 72 h in hACE2-A549 cells.

**FIG 4:**
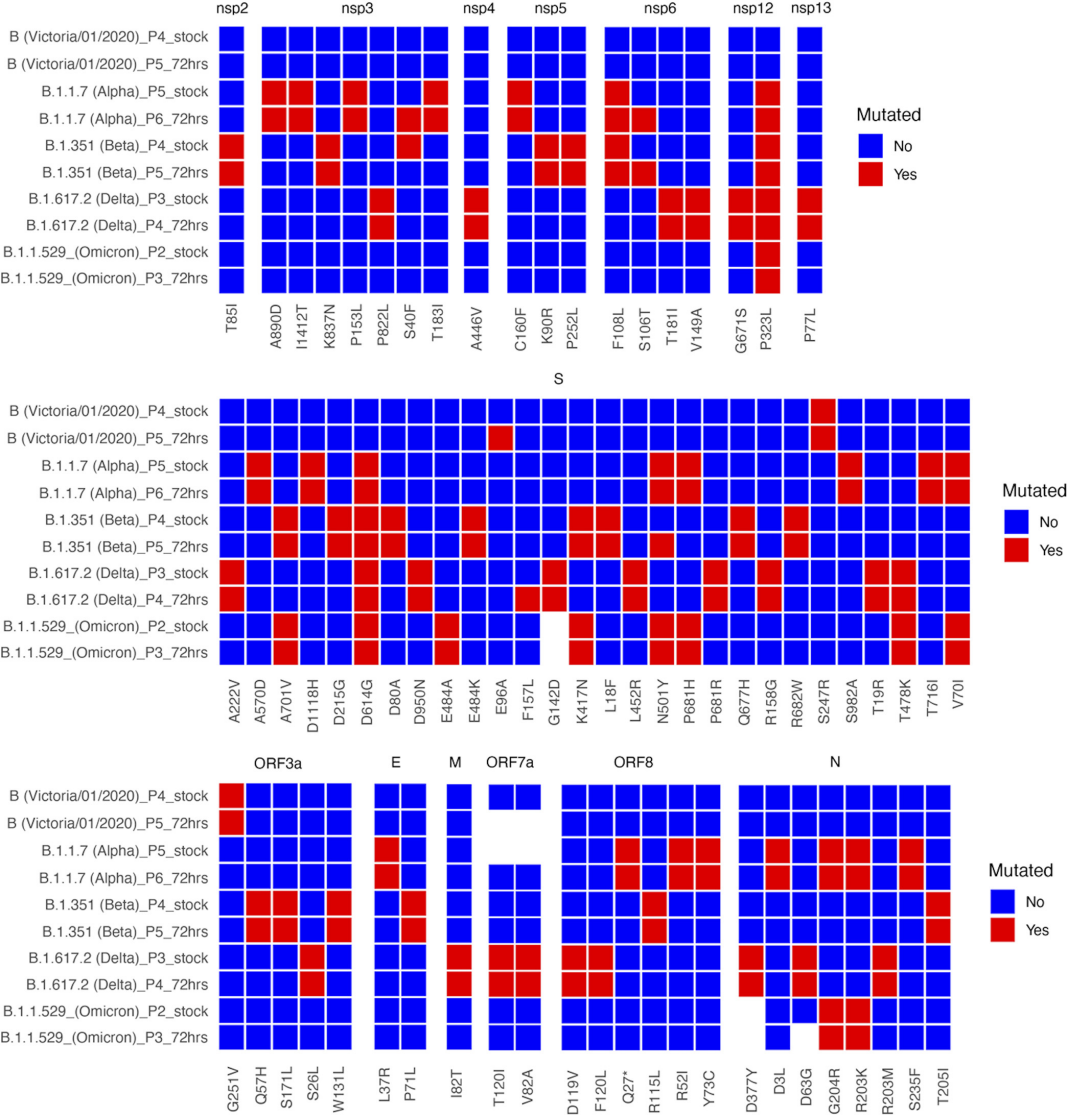
Comparison of stocks (_stock) and supernatant after 72 h of infection in hACE2-A549 cells (_72hrs) of the Victoria/01/2020 isolate (B lineage), the Alpha VOC (B.1.1.7), the Beta VOC (B.1.351), the Delta VOC (B.1.617.2), and the Omicron VOC (B.1.1.529) with the Wuhan reference sequence.

To compare the growth of SARS-CoV-2 from clinical isolates to Alpha, Beta, Delta, and Omicron VOCs, stocks were grown. Growing virus for stocks may have introduced or selected for specific variants. One of those that has been characterized is a deletion of the furin cleavage site in the S gene when grown in Vero E6 cells (21). Therefore, viral stocks were sequenced to ensure they did not possess the deletion and to determine if variation occurred compared to when the virus was sequenced directly from clinical swabs. Comparator variants of known provenance were obtained from collaborators. The comparator variants were the Alpha VOC obtained at P4, SARS-CoV-2/Victoria/01/2020 (a B lineage isolate from Australia) obtained at P3, and, closer to the progenitor virus, the Beta VOC at P4, the Delta VOC obtained at P2, and the Omicron VOC obtained at P2. These were also sequenced to confirm their identity and that stocks did not possess the furin deletion (Fig. 4).

Analysis of the genome diversity between swabs and the virus stock used to infect cells indicated that most dominant viral genome sequence variations from the Wuhan reference sequence were present (Table S1). Minor genomic variants at selected positions were also present. For example, in the stock preparation for SCV2-009, at position 323 in NSP12, this was read with an amino acid depth of 517. The L was present at a depth of 499, P with a depth of 6, and F with a depth of 5, indicating that the dominant viral genome sequence-level amino acid was still present with P and F at a minor level. For some stock viruses, variation from the reference sequence was lost during preparation of the stock virus. The growth of these variants from the stocks was compared to the four VOCs and an ancestral B-lineage SARS-CoV-2/Victoria/01/2020 variant.

### Growth comparison of different SARS-CoV-2 variants to VOCs.

To identify whether the VOCs displayed a growth advantage over contemporary strains of the virus, two different cell lines were infected with the variants at a multiplicity of infection (MOI) of 0.01 over the course of 72 h, and the resultant supernatants at different time points were titrated by plaque assay. The two different cell lines were Vero E6 (commonly used to grow viral stocks of SARS-CoV-2) and hACE2-A549 cells. The latter cell line is a respiratory epithelial cell line that overexpresses the ACE2 protein.

**FIG 5:**
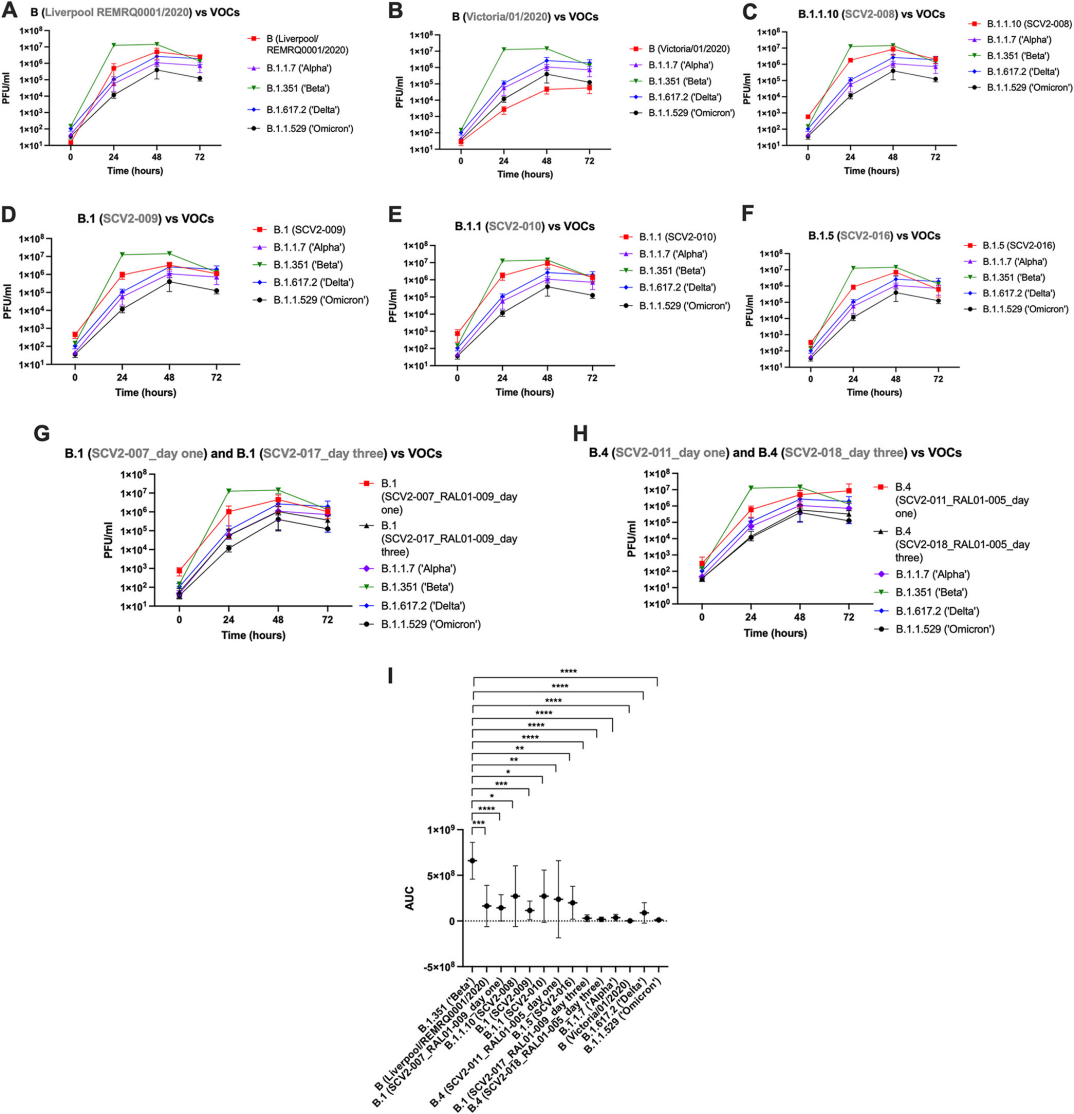
Growth over time of 10 different viral stocks in Vero E6 cells compared to the variants of concern B.1.1.7 (Alpha), B.1.351 (Beta), B.1.617.2 (Delta), and B.1.1.529 (Omicron). (A) Comparison of Liverpool/REMRQ0001/2020 growth against four different VOCs. (B) Comparison of Victoria/01/2020 growth against four different VOCs. (C) Comparison of SCV2-008 against four different VOCs. (D) Comparison of SCV2-009 against four different VOCs. (E) Comparison of SCV2-010 against four different VOCs. (F) Comparison of SCV2-016 against four different VOCs. (G) Comparison of SCV2-007_RAL01-009_day one against SCV2-017_RAL01-009_day three and four different VOCs. (H) Comparison of SCV2-011_RAL01-005_day one against SCV2-018_RAL01-005_day three and four different VOCs. (I) AUC values (±95% confidence intervals) plotted for each variant and compared to the Beta (B.1.351) VOC. All experiments were repeated in triplicate using supernatant from 6 wells (*n* = 3).

In Vero E6 cells a comparison of the growth of each contemporary variant against the VOCs was performed (Fig. 5A to H and Table S2). First, two ancestral B-lineage variants were compared to the VOCS (Fig. 5A and B). After a 1-h attachment period the Liverpool/REMRQ0001/2020 variant grew significantly less than the Beta and Delta VOCs (*P* = 0.002 and *P* = 0.02, respectively). At 24 and 48 h postinfection (hpi), the Liverpool/REMRQ0001/2020 variant did not differ in growth from any VOCs except Beta, which had the highest growth (*P* < 0.0001 and *P* = 0.03, respectively). At 72 h, Liverpool/REMRQ0001/2020 grew at levels similar to those of the Alpha, Beta, and Delta VOCs but more than that of the Omicron variant, the latter of which grew to the lowest levels at 72 h (*P* = 0.03) (Fig. 5A). Another ancestral B lineage variant, the Victoria/01/2020 variant, grew less than all the VOCs, although not significantly at any time point except for the Beta VOC at 0, 24, and 48 hpi (*P* = 0.005, *P* < 0.0001, and *P* = 0.0002, respectively) (Fig. 5B).

10.1128/msphere.00913-21.2 TABLE S2 Vero E6 growth curve statistics. Download Table S2, XLSX file, 0.02 MB. Copyright © 2022 Prince et al. 2022 Prince et al. https://creativecommons.org/licenses/by/4.0/ This content is distributed under the terms of the Creative Commons Attribution 4.0 International license.

Variants with different lineages were then compared to the VOCs. The B.1.1.10 (SCV2-008) isolate grew to higher levels after attachment and 24 hpi than the VOCs but at 48 and 72 hpi grew at levels similar to those of the VOCs (Fig. 5C). The B.1 (SCV2-009) isolate grew significantly more than the Alpha, Delta, and Omicron VOCs (*P* = 0.017, *P* = 0.04, *P* = 0.015, respectively) after attachment but was not different from the Beta VOC. At 24 and 48 hpi, the growth of B.1 (SCV2-009) did not differ from those of the Alpha, Delta, and Omicron VOCs but grew less than the Beta VOC (*P* < 0.0001 and *P* = 0.0023, respectively). By 72 hpi, B.1 (SCV2-009) did not grow differently from any of the VOCs (Fig. 5D).

The growth of the B.1.1 (SCV2-010) isolate was not significantly more than those of the Alpha, Delta, and Omicron VOCs. Compared to the Beta VOC, there was no difference after attachment or at 48 and 72 hpi. However, the growth of the B.1.1 (SCV2-010) isolate was significantly less than that of the Beta VOC at 24 hpi (*P* < 0.0001) (Fig. 5E).

The B.1.5 (SCV2-016) isolate did not grow significantly more than the Alpha, Delta, and Omicron VOCs at 24 and 48 hpi. After attachment the amount of this variant was higher than that of Omicron (*P* = 0.04), and at 24 hpi growth was significantly less than that of the Beta VOC (*P* < 0.0001). By 72 hpi this variant grew to the same levels as the other VOCs (Fig. 5F).

A comparison was then made between the VOCs and variants isolated from the same patient 3 days apart to look for longitudinal differences in variants grown from patients. The twin variants B.1 (SCV2-007_RAL01-009_day one) and B.1 (SCV2-017_RAL01-009_day three) were compared to one another and the VOCs. After attachment there was more B.1 (SCV2-007_RAL01-009_day one) than B.1 (SCV2-017_RAL01-009_day three) (*P* = 0.004) and the other VOCs (*P* = 0.0003, *P* = 0.0013, *P* = 0.0007 and *P* = 0.0003, respectively, for Alpha, Beta, Delta, and Omicron). At 24 and 48 hpi, B.1 (SCV2-007_RAL01-009_day one) grew significantly less than the Beta VOC (*P* < 0.001 and *P* = 0.0037) but was not significantly different from its paired longitudinal sample taken on a different day or any of the VOCs at the other time points (Fig. 5G). Likewise, variants from paired longitudinal samples from another patient, B.4 (SCV2-011_RAL01-005_day one) and B.4 (SCV2-018_RAL01-005_day three), were compared to one another and the other VOCs, but no significant difference was found in the growth of the paired variants at any time point. B.4 (SCV2-011_RAL01-005_day one) grew less than the Beta variant at 24 hpi (*P* < 0.0001) and 48 hpi (*P* = 0.0076) but was not different from the other VOCs. By 72 h, there was no difference between B.4 (SCV2-011_RAL01-005_day one) and any of the VOCs.

Finally, as the Beta VOC appeared to outgrow the other VOCs (Fig. 5A to H), a comparison between VOCs was performed at each time point (Table S2). After attachment there was more Beta than Alpha and Omicron VOCs (*P* = 0.0402 and *P* = 0.0254) but not Delta. The Beta variant grew more than all three other VOCs at 24 hpi (*P* < 0.0001 for each) and at 48 hpi (*P* = 0.0021, *P* = 0.0045 and *P* = 0.0015 for Alpha, Delta, and Omicron, respectively). However, at 72 hpi, there was no significant difference between the growth of VOCs in Vero E6 cells. While it appeared that Omicron grew less well than Alpha and Delta, this was not statistically significant at any of the time points (Fig. 5A to H). An area under the curve (AUC) analysis also confirmed the Beta variant appeared to grow differently from all variants including the VOCs (Fig. 5I and Table S2).

**FIG 6:**
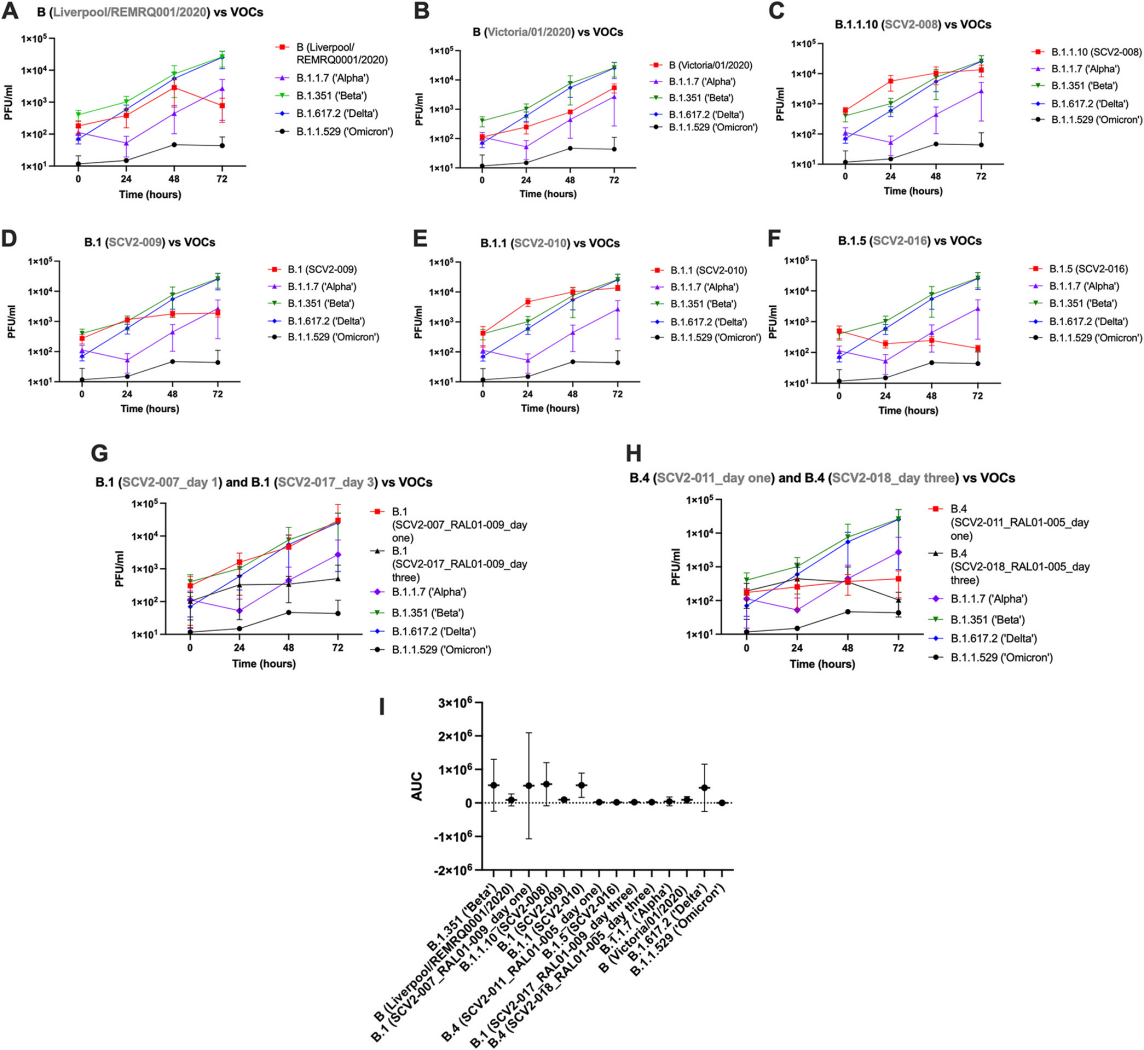
Growth over time of 10 different viral stocks in hACE2-A549 cells compared to the variants of concern B.1.1.7 (Alpha), B.1.351 (Beta), B.1.617.2 (Delta), and B.1.1.529 (Omicron). (A) Comparison of Liverpool/REMRQ0001/2020 growth against four different VOCs. (B) Comparison of Victoria/01/2020 growth against four different VOCs. (C) Comparison of SCV2-008 against four different VOCs. (D) Comparison of SCV2-009 against four different VOCs. (E) Comparison of SCV2-010 against four different VOCs. (F) Comparison of SCV2-016 against four different VOCs. (G) Comparison of SCV2-007_RAL01-009_day one against SCV2-017_RAL01-009_day three and four different VOCs. (H) Comparison of SCV2-011_RAL01-005_day one against SCV2-018_RAL01-005_day three and four different VOCs. (I) AUC values (±95% confidence intervals) plotted for each variant and compared to the Beta (B.1.351) VOC. All experiments were repeated in triplicate using supernatant from 6 wells (*n* = 3).

In contrast, in hACE2-A549 cells, there was more heterogeneity observed between variants, with the range of viral titers being much lower than that observed in Vero cells (Fig. 6A to H and Table S3). Examining the ancestral B lineage variants did not yield any significant differences in growth. The growth of Liverpool/REMRQ0001/2020 did not differ significantly from that of any of the VOCs at any of the time points (Fig. 6A). There was less ancestral virus (Victoria/01/2020) than the Beta VOC after attachment (*P* = 0.029) but no significant difference in the amount of virus at any other time points compared to any of the VOCs (Fig. 6B).

10.1128/msphere.00913-21.3 TABLE S3 Human ACE2-A549 growth curve statistics. Download Table S3, XLSX file, 0.02 MB. Copyright © 2022 Prince et al. 2022 Prince et al. https://creativecommons.org/licenses/by/4.0/ This content is distributed under the terms of the Creative Commons Attribution 4.0 International license.

There was more B.1.1.10 (SCV2-008) variant than Alpha, Delta, and Omicron VOCs after attachment (*P* = 0.0187, *P* = 0.0189, and *P* = 0.0096, respectively). There was no significant difference between this variant and any of the VOCs at the other time points (Fig. 6C). The B.1 (SCV2-009) variant only differed in growth from the VOCs at 24 hpi, where it grew more than Alpha (*P* = 0.0117) and Omicron (*P* = 0.0164) (Fig. 6D). The B.1.1 (SCV2-010) variant differed from the VOCs only in growth at 24 hpi, where it grew more than the Alpha, Beta, Delta, and Omicron VOCs (*P* = 0.0029, *P* = 0.0233, *P* = 0.0119, and *P* = 0.0048, respectively) (Fig. 6E and Table S3). The B.1.5 (SCV2-016) variant did not grow significantly differently from the other VOCs except at 24 hpi, where it grew significantly less than the Beta VOC (*P* = 0.0341) (Fig. 6F).

B.1 (SCV2-007_RAL01-009_day one) was compared to virus isolated from its paired longitudinal sample, B.1 (SCV2-017_RAL01-009_day three), in addition to the VOCs. There was no significant difference between growth of the paired variants at any time point, and the only difference between B.1 (SCV2-007_RAL01-009_day one) and the VOCs was at 24 hpi, where it grew significantly more than Alpha (*P* = 0.0387) (Fig. 6G). Likewise, the B.4 (SCV2-011_RAL01-005_day one) variant was compared to its paired variant B.4 (SCV2-018_RAL01-005_day three) and the VOCs, and no significant difference in growth was observed at any time point (Fig. 6H).

Finally, the growth levels of the VOCs were compared to one another. After attachment there was more Beta than Alpha (*P* = 0.0402) and Omicron (*P* = 0.0254), while at 24 and 48 hpi there was more Beta than Alpha (*P* < 0.0001 and *P* = 0.0021, respectively), Delta (*P* < 0.0001 and *P* = 0.0045, respectively), and Omicron (*P* < 0.0001 and *P* = 0.0015, respectively). However, by 72 hpi there was no difference in growth between the VOCs (Table S3). This was confirmed by an AUC analysis, which found no significant difference between the growth of Beta and any of the other variants (Fig. 6I).

### The phenotype of the variants differed widely between cell lines, displaying mixed plaque morphology and growth characteristics.

**FIG 7:**
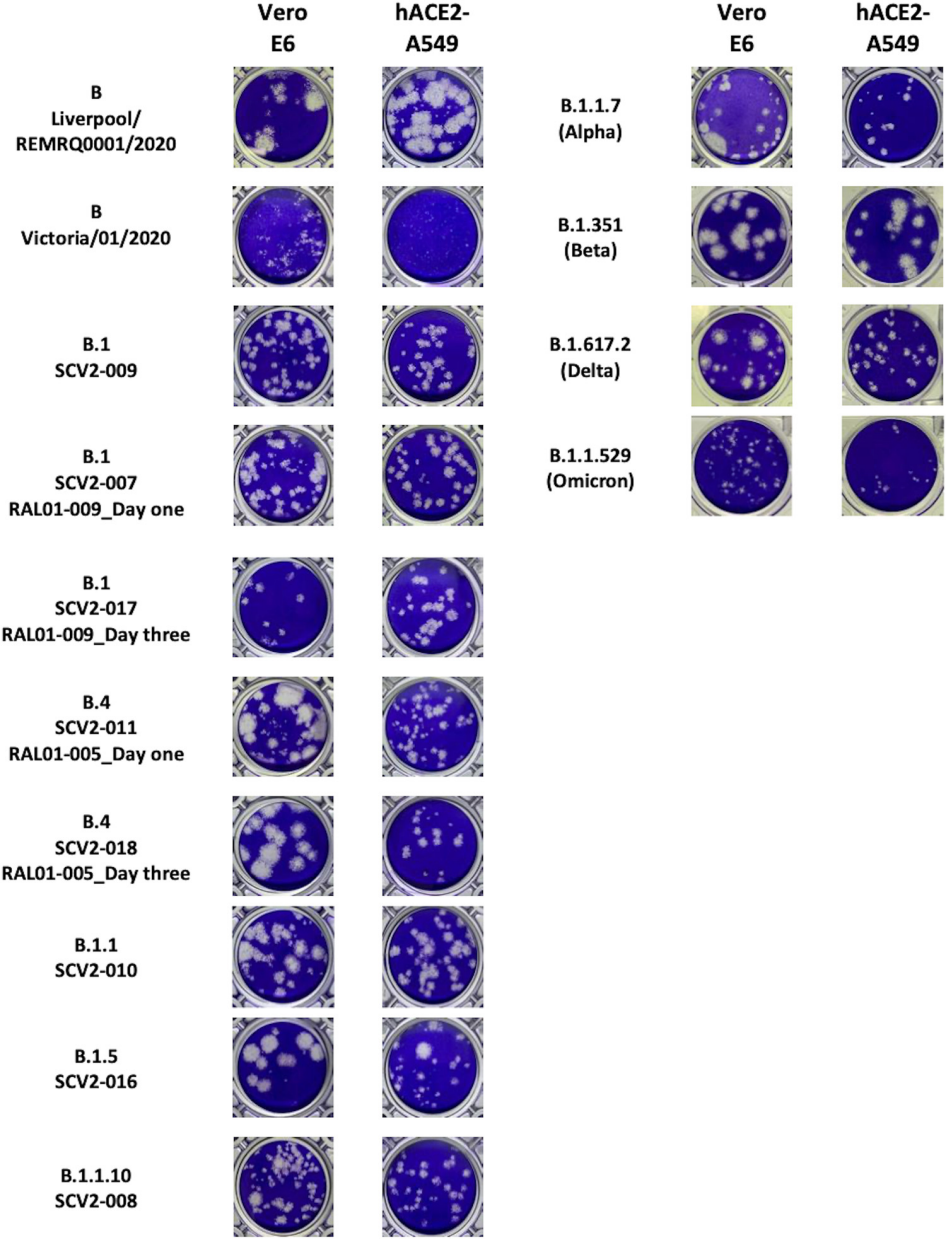
Phenotypic appearance of plaque assays from variants grown in two different cell lines for 72 h. i, Vero E6 cells; ii, hACE2-A549 cells. Plaque assays were performed on Vero E6 cells. Variants of concern are B.1.1.7 (Alpha VOC), B.1.351 (Beta VOC), B.1.617.2 (Delta VOC), and B.1.1.529 (Omicron VOC). Comparison variants include the Victoria/01/2020 and the Liverpool/REMRQ0001/2020 variants.

**FIG 8:**
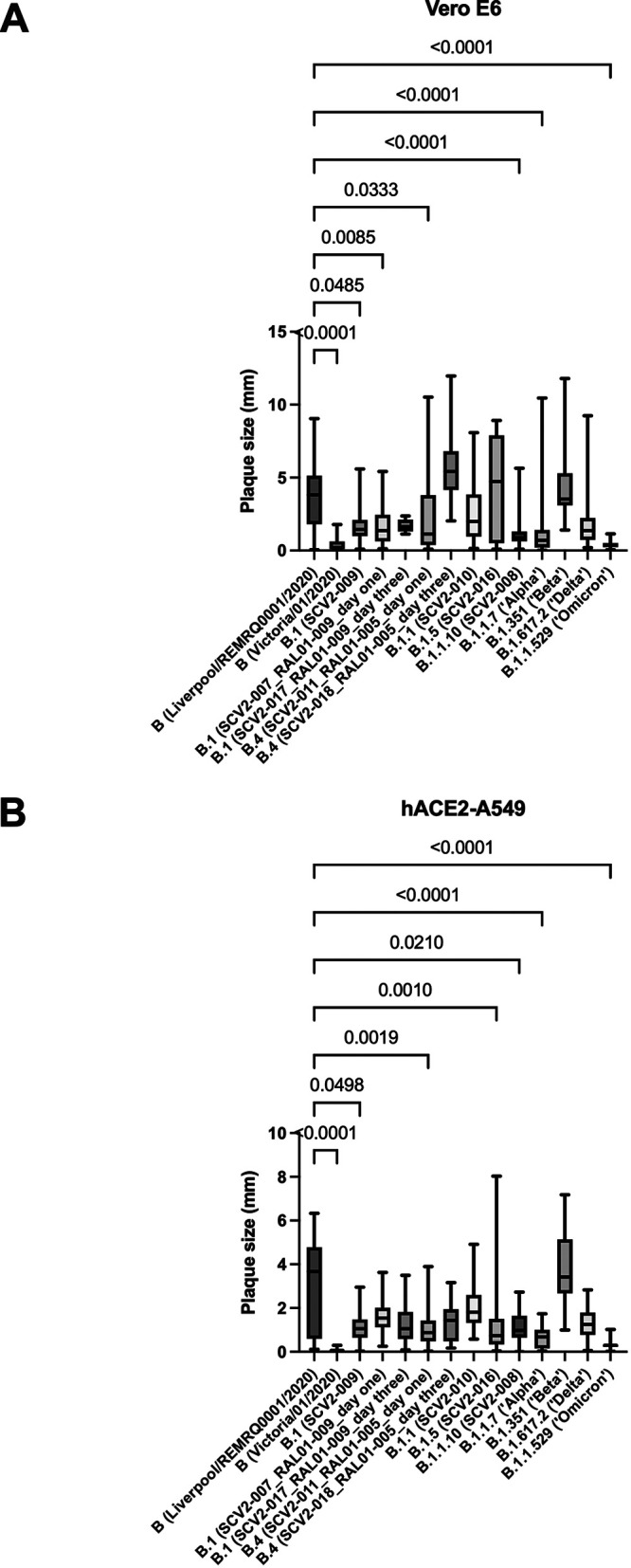
Plaque area sizes for variants grown at 72 h postinfection in Vero E6 cells (A) and hACE2-A549 cells (B). Plaque sizes were measured using Image J and plotted to demonstrate the spread and variance in plaque sizes. Sizes are shown in square millimeters.

The phenotype of the plaques formed by supernatants generated at 72 h postinfection for both cell lines was characterized. This time point was chosen as it provided the virus maximum time to replicate in the cell line and allow generation of virus for sequencing. The morphology of the plaques between the variants differed (Fig. 7 and 8). After growth in Vero E6 cells, the Liverpool/REMRQ-0001/2020, SCV2-011_RAL01-005_day one, SCV2-016, SCV2-018_RAL01-005_day three, and Beta variants had larger plaque phenotypes than SCV2-008, SCV2-009, SCV2-007_RAL01-009_day one, SCV2-011_RAL01-005_day one, Alpha, Omicron, and SARS-CoV-2/Victoria/01/2020 variants (Fig. 8). Equally, some variants displayed a mixed phenotype of both large and small plaques, as seen for SCV2-011_RAL01-005_day one and B.1.617.2 (Delta), suggesting slightly different variants were present (Fig. 7 and 8). This observation has also been recently described (22).

After growth in hACE2-A549 cells for 72 h, the Liverpool/REMRQ-0001/2020 variant and the Beta VOC had the largest plaque phenotypes, while the SARS-CoV-2/Victoria/01/2020 and the Omicron variants had the smallest plaque phenotypes (Fig. 7 and 8). The Liverpool/REMRQ-0001/2020, B.1.5 (SCV2-016), and Beta variants had both large and small plaques. This illustrates the potential diversity with a viral population. A comparison between the median plaque sizes of Liverpool/REMRQ-0001/2020 and the other variants found it had significantly larger plaques than B (Victoria/01/2020), B.1 (SCV2-009), B.4 (SCV2-011_RAL01-005_day one), B.1.5 (SCV2-016), B.1.1.10 (SCV2-008), Alpha, and Omicron variants (Fig. 8).

### Genetic diversity of variants after passage in hACE2-A549 cells.

We hypothesized that differences in the phenotypic appearance of variants and their reproduction reflect the presence of minor genomic variants and stop codons in their underlying sequences. This was investigated by sequencing the supernatants generated at 72 h in hACE2-A549 cells from growth assays, also used in phenotype assays (Fig. 1). All variants have a dominant virus-level genome but also minor genomic variants. In SCV2-016, at 72 h postinfection there was a stop codon at dominant viral genome sequence level in ORF3A that was also present in the viral stock (Table S1). We note that this was sequenced with low read depth, and other amino acids were present at the minor genomic variant levels. Stop codons were also present in the variants at a minor genomic variant level (Table S1).

## DISCUSSION

Sequence analysis of SARS-CoV-2 in clinical swabs from patients with COVID-19 revealed a heterogenous and diverse population from the Wuhan reference sequence, as previously described (23). Examples of substitutions within viral populations in patients include those caused by C-to-U and A-to-G transitions but also some examples of the more infrequent transversions, such as G to C (see Table S1 in the supplemental material). Hypermutation, possibly as a result of ADAR- and APOBEC-associated activity, has been noted for SARS-CoV-2 (24) and Middle East respiratory syndrome-CoV (25). Potential modification of coronavirus genomes by cellular processes has been associated with restricting the growth of human coronavirus NL63 (HCoV-NL63) (26).

At the minor genomic variant level, several variants had genomes that contained premature stop codons. For example, the B.1.1 variant, SCV2-010, differs from the reference sequence at position 204 in the N protein by an R instead of a G but also possessed a stop codon at the minor genomic variant level. In stocks, this stop codon was maintained at the minor genomic variant level; however, it was lost upon culture in hACE2-A549 cells for 72 h and replaced by a Q. Examples of SARS-CoV-2 genomes encoding nonfunctioning proteins have been previously identified in the human population. For example, a cohort of patients in Singapore was identified with a deletion in ORF8 that was associated with a milder infection (27), although the variant disappeared either through control measures or lack of fitness. A limitation of using a nondirect sequencing approach to identify minor genomic variants is that changes can be due to errors during sequencing, although these would likely be at a background level. To balance this, sequence quality and depth of coverage were considered in this analysis, and the ARTIC pipeline includes a strict filtration step to help mitigate this.

The potential disconnect between the dominant viral genome sequence and minor genomic variants with potentially different phenotypes in a patient is not restricted to SARS-CoV-2. The balance between the genomes and the presence of stop codons in variant populations within individual patients has been shown to influence the global activity of the Ebola virus RNA-dependent RNA polymerase and correlate with outcome in patients with Ebola virus disease (28). Within an individual with SARS-CoV-2, these mixtures of functioning and presumably nonfunctioning viral proteins potentially will influence viral load and interactions with host cell signaling pathways.

The growth of different variants, and a variant from near the start of the COVID-19 pandemic that lacked D614G, were compared in two different cell lines to the growth of the Alpha, Beta, Delta, and Omicron VOCs, which represent variants that have an apparent transmission advantage and/or may be less refractive to currently approved vaccines.

In Vero E6 cells, the growth of two ancestral B-lineage variants were compared to the VOCs. The Liverpool/REMRQ0001/2020 variant grew no differently from Alpha at any time point, grew significantly less than Beta at 24 to 48 h, and grew more than Omicron at 72 h. In contrast, the other B-lineage variant, Victoria/01/2020, appeared to grow less than all the VOCs, although this was not statistically significant at any time point. In hACE2-A549 cells there was much more heterogeneity in growth between the different variants, with no significant difference being observed between the Liverpool/REMRQ0001/2020 and the VOCs, while Victoria/01/2020 grew significantly less than Beta but not significantly differently than Alpha, Delta, and Omicron. The increased heterogeneity observed in hACE2-A549 cells most likely was due to the production of interferon by these cells, whereas Vero E6 cells do not secrete interferon, so variants may replicate without interference. Interestingly, sequencing revealed the Victoria/01/2020 variant had only 3 mutations from the Wuhan reference, S-E96A, S-247R, and ORF3a-G251V, while the Liverpool/REMRQ0001/2020 variant had N-R203K, NSP6-L37F, NSP3-N1410T, and NSP16-A116V. The R203K mutation has been noted to appear alongside G204R mutation as seen in the B.1.1.7 (Alpha) VOC and has been associated with increased infectivity and fitness (29). It is possible that the presence of this mutation in the Liverpool/REMRQ0001/2020 variant caused slightly better growth than the Victoria/01/2020 variant in Vero E6 cells. In contrast, the L37F mutation in NSP6 has been suggested to correlate with asymptomatic infection and reduced virulence (30).

In Vero E6 cells, the B.1.1.10 (SCV2-008), B.1 (SCV2-009), B.1.1 (SCV2-010), and B.1.5 (SCV2-016) variants appeared to grow comparably but with small differences. For example, there was more B.1.1.10 (SCV2-008) than all the VOCs after attachment, while there was no distinction between B.1.1 (SCV2-010) at this time point against the VOCs. By 72 hpi, the growth of these variants was not significantly different from the VOCs. This pattern was repeated in hACE2-A549s, where there was no difference in growth between these variants at 48 or 72 hpi. The largest difference in growth between isolates and the VOCs in hACE2-A549 cells was observed at 24 hpi. We propose that this is due to the replication time for SARS-CoV-2; by 72 hpi all cells had been infected and extensive cytopathic effect (CPE) was observed in Vero E6 cells (hACE2-A549 cells do not display CPE). While SARS-CoV-2 is thought to exit the cell through continuous budding rather than being lytic (31), it is possible that by 72 hpi the virus has exhausted the ability of the cells to continuously manufacture new viruses at the same rate. Others groups have suggested time from absorption to release of new viral particles is between 8 (32) and 36 (33) hpi, which would explain the differences observed at 24 hpi. We propose that exploring time points after 24 hpi is not useful for detecting differences in growth. Furthermore, hACE2-A549 cells secrete interferon and therefore could explain the increased variance seen in this cell line at 24 hpi.

The growth of variants isolated from patients 3 days apart was also evaluated. In both cell lines, the paired variants did not differ significantly in amount except after attachment between B.1 (SCV2-007_RAL01-009_day one) and B.1 (SCV2-017_RAL01-009_day three) in Vero E6 cells. Sequencing analysis demonstrated that B.1 (SCV2-007_RAL01-009_day one) had more variations from the reference at the dominant viral genome sequence level than its paired variant, B.1 (SCV2-017_RAL01-009_day three). However, most nucleotide changes reverted 3 days later in cell culture. This suggests minimal intrahost variation at least early during infection, although increased variation has been observed over longer periods (34, 35).

A comparison between the VOCs found that Beta exceeded the growth of the other variants at 24 and 48 hpi but was not significantly different at 72 hpi in both cell lines. An area under the curve (AUC) analysis found the Beta VOC grew significantly differently from all other variants in Vero E6 cells; however, this was not the case in hACE2-A549s, likely because of the increased heterogeneity observed. This result has been replicated in a recent publication comparing infection of different VOCs in Vero E6 cells (22) and may be due to a shorter eclipse phase during Beta replication (36). Our results contrast with a study using pseudotyped virus, where the data suggested that the Beta VOC was less infectious than Delta (37). This may be due to differences in biology between pseudotyped virus and live virus and/or that variations in other viral proteins influence growth. The Beta variant grown in this study had both the Q57H substitution in ORF3a and the T85I substitution in NSP2, mutations previously posited to cause reduced growth in Vero CCL81 cells (38). However, under the conditions used in our study, no significant inhibition of growth compared to other variants was observed. This indicates differences in cell lines used to evaluate viral growth *in vitro*.

The Alpha (B.1.1.7) VOC did not grow significantly more than any of the other VOCs or clinical isolates. Extrapolating this observation to the perceived transmission advantage that B.1.1.7 had in the human population at the time, the data suggest this was not down to the VOC growing to higher titers in cells than with other variants. However, we acknowledge that *in vitro* growth comparisons may not correlate exactly with growth rates *in vivo*. These *in vitro* properties of B.1.1.7 have been noted before from different clinical isolates (22, 39, 40). However, in Calu-3 cells, another human lung cell line, B.1.1.7 and B.1.351 were found to have similar growth rates (41), demonstrating differences observed between cell lines, isolates, and MOIs used. Alpha has been suggested to possess an advantage through suppression of the interferon response to viral infection (42).

The Omicron VOC has been shown to outgrow Delta in human upper airway cells (43) at 24 hpi but not at later time points. In published data (43), the growth of Omicron in Vero-AT cells did not differ from Delta, while in Calu-3 cells Omicron was found to grow to lower titers than the Delta VOC. In our study, the growth of Omicron, while not significant, did appear to grow to lower titers than the other VOCs in hACE2-A549s. The Omicron VOC is thought to enter cells in a predominantly TMPRSS2-independent fashion via the endosomal route, where viruses are exposed to IFTIM restriction factors (43). Vero E6 cells lack both the TMPRSS2 protease and interferon, so perhaps it is not surprising that the growth of Omicron did not differ significantly from the other VOCs in this cell line. Human ACE2-A549 cells also lack the TMPRSS2 protease, so all the variants used would need to employ the endosomal route of entry. The Omicron VOC replicates much better in the upper airway than the lower airway in *ex vivo* models (44) and *in vivo* models (45, 46). Therefore, the lower titers of virus observed in hACE-A549 may be a result of their alveolar origin or genetic differences between the cell line and normal human cells.

The Liverpool/REMRQ-0001/2020 and Beta variants had the largest plaque phenotypes after growth in both cell lines, followed by Delta, Alpha, and Omicron. The large plaque size of Beta has been noted before in Vero E6 cells (36, 47). One argument is that the large plaque size correlates with the thermal stability of the virus (22). Others suggest that the 614G-501Y-484K-417N combination of mutations in Beta allow increased attachment to ACE2, entry, and replication compared to other VOCs (47).

The smaller phenotype of the Alpha (B.1.1.7) variant has also been noted in other studies too (39). The smallest plaques were found with the Omicron (in Vero E6) and Victoria/01/2020 (in hACE2-A549) variants. Current research suggests that the Alpha, Beta, and Delta VOCs have improved ability to form syncytia in culture, while Omicron is thought to have impaired fusogenicity in culture and thus smaller plaques (41, 48, 49).

As studies differ in their findings comparing the growth properties of VOCS (42, 50), and as we only tested one isolate each of the four VOCs, it is similarly possible that our results are due to isolate-specific properties. One of the deficiencies of performing such research is that many studies do not elaborate on the passage number of the viruses used and use different cell lines and MOIs, complicating efforts to compare and standardize results. A limitation of this study was the use of variants at different passages, as we were not able to isolate all the variants ourselves and relied on the kind donation of variants. This illustrates the difficulties in studying variants during the pandemic when time is of the essence and a central resource for supply of virus for *in vitro* studies may not be available. Others groups have shown that growth in cell culture can introduce changes in the sequence of SARS-CoV-2 (51), although we did not observe significant changes with passage of the virus in this study.

The analysis of virus in the nasopharyngeal swabs clearly paints a picture of a diverse population of SARS-CoV-2. However, in contrast to SARS-CoV-2, different variants of the coronavirus infectious bronchitis virus (IBV) can individually cause different spectrums of organ specific disease, and therefore current variations in the genome of SARS-CoV-2 may not automatically equate to radically different disease, as observed with IBV. Due to the promiscuous nature of coronavirus RNA synthesis, variants have and will occur all the time. This emphasizes the need for genotype-to-phenotype studies to place newly emerged variants that have perceived differences in context.

When studying isolates, even when grown in cell culture, population diversity continues. Thus, while lineage-defining variations are present at a dominant viral genome sequence level, minor genomic variants are present underneath that may have an impact on biology. This implies that the study of specific genotypes requires either plaque purification or reverse genetics. We propose that the viral population should be considered when studying the transmission of SARS-CoV-2, as ultimately, in patients, the virus exists as a population.

## MATERIALS AND METHODS

### Cells.

African green monkey kidney C1008 (Vero E6) cells (Public Health England, PHE) were cultured in Dulbecco’s minimal essential medium (DMEM) (Sigma) with 10% fetal bovine serum (FBS) (Sigma) and 0.05 mg/mL gentamicin at 37°C and 5% CO_2_. Vero/hSLAM cells (PHE) were grown in DMEM with 10% FBS and 0.05 mg/mL gentamicin (Merck) with the addition of 0.4 mg/mL Geneticin (G418; Thermofisher) at 37°C and 5% CO_2_. Human ACE2-A549 (hACE2-A549), a lung epithelial cell line that overexpresses the ACE2 receptor, was the kind gift of Oliver Schwartz (52) and cultured in DMEM with 10% FBS and 0.05 mg/mL gentamicin with the addition of 10 μg/mL Blasticidin (Invitrogen). Only passage 3 to 10 cultures were used for experiments.

### Virus isolation.

The SARS-CoV-2/human/Liverpool/REMRQ0001/2020 isolate (GenBank accession no. MW041156.1) was obtained at passage 3. The fourth passage of virus was cultured in Vero E6 cells with DMEM containing 4% FBS and 0.05 mg/mL gentamicin at 37°C and 5% CO_2_ and harvested 48 h postinoculation. Virus stocks were aliquoted and stored at −80°C.

Variants named SCV2-007 to SCV2-018 were grown from nasopharyngeal swabs of patients using the following method. One hundred microliters of viral transport medium from the swab was mixed with 100 μL DMEM with 4% FBS, 0.05 mg/mL gentamicin, 25 μg/mL plasmocin (Invivogen), and 2.5 μg/mL amphotericin B (Merck). These were then filtered using ultrapure MC 0.22-μm filters (Merck) and the filtrate placed onto cells in a 24-well plate of Vero E6 cells for 1 h. After 1 h, the medium was topped up with DMEM (2% FBS, 0.05 mg/mL gentamicin, 25 μg/mL plasmocin, 2.5 μg/mL amphotericin B). Cells were observed daily for cytopathic effect (CPE), and the cell supernatant was harvested once CPE was evident. This provided the first-passage virus. Stocks of these were then grown in Vero E6 as described above, frozen in aliquots at −80°C, and named SCV2-007 to SCV2-018_stockP2.

The B.1.1.7 and B.1.351 isolates were obtained at passage 4. The fifth-passage stocks were cultured in Vero/hSLAM cells with DMEM containing 4% FBS, 0.05 mg/mL gentamicin, and 0.4 mg/mL Geneticin and harvested 72 h postinoculation. Virus stocks were aliquoted and stored at −80°C. The B.1.617.2 and B.1.1.529 isolates were obtained at passage 2 and the third-passage stocks cultured in Vero/hSLAM cells as described above. SARS-CoV-2 Victoria/01/2020 was passaged three times in Vero/hSLAM cells. The fourth-passage stock was cultured in Vero/hSLAM cells in DMEM containing 4% FBS, 0.05 mg/mL gentamicin, and 0.4 mg/mL Geneticin and harvested 72 h postinoculation. Virus stocks were aliquoted and stored at −80°C (Table 1).

### Virus titration.

Viral titers of stocks were calculated using plaque assays. Briefly, confluent 24-well plates of Vero E6 cells were inoculated with serial 10-fold dilutions of the stocks in duplicate for 1 h at 37°C and 5% CO_2_. Plates were overlaid with DMEM containing 2% FBS, 0.05 mg/mL gentamicin, and 2% low-melting-point agarose (Lonza) and incubated at 37°C and 5% CO_2_ for 72 h. Plates were fixed using 10% formalin, the overlay removed, and plates stained using crystal violet solution (Sigma). Virus titer was measured in number of PFU per milliliter.

### Virus growth kinetics.

Vero E6 and hACE2-A549 cells were grown in 96-well plates for viral growth kinetic experiments. For infection, medium was removed from plates and virus inoculum added at an MOI of 0.01 in DMEM containing 2% FBS, 0.05 mg/mL gentamicin, and the respective selective antibiotics for each cell line (6 wells per time point). Plates were incubated at 37°C and 5% CO_2_ for 1 h. The inoculum was removed and cells were washed once with PBS (Sigma). The respective medium with 2% FBS (100 μL) was added to each well. The cell supernatant was removed from wells and combined (0 h postinfection), and plates were incubated further. Supernatants were likewise removed at 24, 48, and 72 h postinfection. Approximately 250 μL of the supernatants was aliquoted directly into tubes containing 750 μL TRIzol LS (Fisher) to inactivate the virus. All supernatants and inactivated supernatants were stored at −80°C until viral titration and RNA extraction could be performed. All infections were performed at least three times in independent experiments.

### RNA extraction and amplification of viral nucleic acids.

RNA from clinical samples was extracted and DNase treated as described previously. Samples from patients were sequenced using the RLSA approach (53). RNAs from viral stocks and from 72-h postinfection cultures were sequenced by Oxford Nanopore long-read-length sequencing on flow cells run on MinION or GridION.

### Nanopore sequencing.

Sequencing libraries for amplicons generated by RSLA (53) or ARTIC were prepared by following the PCR tiling of SARS-CoV-2 virus with the Native Barcoding protocol provided by Oxford Nanopore Technologies using LSK109 and EXP-NBD104/114.

### Variant calling.

The artic-ncov2019 pipeline v1.2.1 (https://artic.network/ncov-2019/ncov2019-bioinformatics-sop.html) was used to filter the passed Fastq files produced by Nanopore sequencing with lengths between 800 and 1,600 for RSLA and 400 and 700 for ARTIC. This pipeline was then used to map the filtered reads on the reference SARS-CoV-2 genome (NC_045512.2) by minimap2, assigned each read alignment to a derived amplicon, and excluded primer sequences based on the RSLA and ARTIC V3 primer schemes in the bam files. These bam files were further analyzed using DiversiTools (http://josephhughes.github.io/DiversiTools/) with the “-orfs” function to generate the ratio of amino acid change in the reads and coverage at each site of protein compared to the reference SARS-CoV-2 genome (NC_045512.2). The amino acids with highest ratio and coverage of >10 were used to assemble the dominant viral protein sequences.

### Phlyogenetic analysis.

Phylogenetic analysis comprised the dominant viral genomes from this study with the Wuhan strain (NC_045512.2) as the outgroup. The genomes were partitioned into four sets of sites: 1st, 2nd, and 3rd codon positions of the protein-coding regions and the noncoding intergenic regions. PartitionFinder (v2.1.1) (54) was used to select partitioned models of evolution for phylogenetic analyses in these four sets of sites with default settings. The selected models were used to construct a Bayesian nucleotide divergence tree using MrBayes (v3.2.7) (55).

### Measurement of plaque sizes.

A minimum of 10 plaques from each replicate were measured using Image J and the results expressed in square millimeters.

### Statistics.

Viral titers at each time point were compared using one-way analysis of variance (ANOVA) with correcting for multiple comparisons using *post hoc* Tukey tests. In addition, an AUC analysis was used to compare all variants across all time points and then a one-way ANOVA with correcting for multiple comparisons using the *post hoc* Tukey test was used to compare AUC values. Comparison of plaque sizes was performed using the Kruskal-Wallis test. Results were considered significant at a *P* value of <0.05. All statistics were performed using GraphPad Prism v9.3.1.

### Ethics and clinical information.

The patients from whom the virus samples were obtained gave informed consent and were recruited under the International Severe Acute Respiratory and emerging Infection Consortium (ISARIC) WHO Clinical Characterization Protocol CCP. Ethical approval for data collection and analysis by ISARIC4C was given by the South Central-Oxford C Research Ethics Committee in England (reference 13/SC/0149) and by the Scotland A Research Ethics Committee (reference 20/SS/0028). Samples were use with consent from patients or consultees. The ISARIC WHO CCP-UK study was registered at https://www.isrctn.com/ISRCTN66726260 and designated an Urgent Public Health Research Study by NIHR. Protocol, patient information sheets, consents, case report forms, and process of data and sample access request are available at https://ISARIC4C.net.

### Biosafety.

All work was performed in accordance with risk assessments and standard operating procedures approved by the University of Liverpool Biohazards Subcommittee and by the UK Health and Safety Executive. Work with SARS-CoV-2 was performed at containment level 3 by personnel equipped with respirator airstream units with filtered air supply.
